# Non-aneurysmal Subarachnoid Hemorrhage in an Adult With Sickle Cell Anemia: A Case Report and Review of the Literature

**DOI:** 10.7759/cureus.101929

**Published:** 2026-01-20

**Authors:** Fahad M Okal, Tala AlSindi, Naif F AlHarbi, Khalid M Bajunaid

**Affiliations:** 1 Neurosurgery, King Abdulaziz Medical City, Jeddah, SAU; 2 Neurosurgery, King Abdulaziz University Hospital, Jeddah, SAU; 3 Neurology, King Fahad General Hospital, Jeddah, SAU; 4 Vascular Neurosurgery, King Abdulaziz University Hospital, Jeddah, SAU

**Keywords:** aneurysm, collateral, moyamoya disease, moyamoya syndrome, subarachnoid hemorrhage

## Abstract

Moyamoya disease (MMD) is a rare, idiopathic cerebrovascular disorder characterized by bilateral stenosis of the internal carotid arteries (ICAs) and the formation of collateral vessels at the base of the brain. In patients with sickle cell disease (SCD), chronic vaso-occlusive changes can produce moyamoya-like vasculopathy, resulting in moyamoya syndrome (MMS) and increasing the risk of aneurysm formation and rupture. Nonetheless, presentation of MMS as non-aneurysmal subarachnoid hemorrhage (NASAH) is extremely rare, with few reported cases.

Our case is about a 28-year-old male, a known case of SCD, who developed NASAH. He presented with a sudden decrease in consciousness, severe headache, nausea, and vomiting. Emergency evaluation revealed significant neurological impairment. Unenhanced brain computed tomographic imaging showed pure intraventricular hemorrhage. Computed tomographic angiography revealed the absence of contrast filling in both ICAs at the level of bifurcation, with prominent posterior circulation. Suspicion of MMS was raised. Diagnostic cerebral angiography revealed a complete absence of the right ICA and an almost completely occluded left ICA terminating at the anterior communicating artery (Acom). A run-through vertebral artery injection revealed the classic “puff of smoke” appearance, confirming the diagnosis of MMS. The patient was managed conservatively with the SAH protocol and was admitted to the intensive care unit. The patient passed away after a complicated course with multiple stroke events. MMD presenting as NASAH is uncommon and is believed to result from rupture of fragile vessels associated with MMD. This case supports previous findings, highlighting the importance of recognizing this presentation for timely management.

## Introduction

Moyamoya disease (MMD) is an uncommon cerebrovascular disease that is considered idiopathic. It is described as a progressive, nonatherosclerotic, steno-occlusive disease of the internal carotid arteries (ICAs) bilaterally, along with the formation of an abnormal network of vessels, or collaterals, at the base of the brain, which explains the term “moyamoya,” or “puff of smoke,” in Japanese [1].

The incidence of MMD varies considerably according to geographic distribution. A high incidence has been reported in East Asia, particularly in Japan and South Korea, with an annual incidence of 1.7 to 2.3 per 100000 and an average annual prevalence of 19.1% [2]. It is less common in Western countries [3]. Globally, the age of onset for MMD peaks in the first decade of life, with a moderate peak in the third and fourth decades of life [4]. The classic presentation of MMD varies according to the age of onset. In children, MMD is responsible for up to 6% of childhood strokes, including ischemic strokes and transient ischemic attacks (TIAs), whereas hemorrhage, either intracerebral hemorrhage (ICH), intraventricular hemorrhage (IVH), or rarely subarachnoid hemorrhage (SAH), has been described in adults [5,6].

As sickle cell disease is a multisystem disease, it can complicate intracranial vascular pathology. Chronic vaso-occlusive changes can produce moyamoya-like vasculopathy, which is known as moyamoya syndrome (MMS). It can have an aggressive natural history, including an increased risk of aneurysm formation and rupture [7]. Nonetheless, presentation of MMS as non-aneurysmal SAH (NASAH) is extremely rare, with few reported cases [8-16].

Here, we report the case of a 28-year-old male with known sickle cell disease who developed NASAH with unusual angiographic findings.

## Case presentation

**Figure 1:**
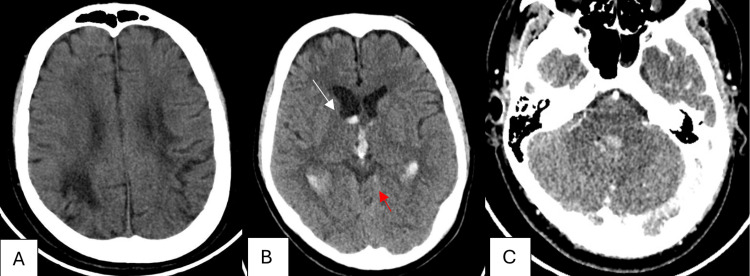
(A, B) Unenhanced brain CT and (C) contrast-enhanced brain CT showing IVH (white arrow) with minimal SAH (red arrow), along with previously known right and left parietal infarctions SAH: subarachnoid hemorrhage; IVH: intraventricular hemorrhage; CT: computed tomography

A 28-year-old male with a known history of sickle cell disease (SCD) presented to the emergency department (ED) via the Red Crescent with a sudden decrease in the level of consciousness, preceded by a severe headache associated with nausea and vomiting. On emergency assessment, he was tachycardic and agitated. His pupils were equally reactive. The right lower limb demonstrated clonus with increased Achilles tendon and knee reflexes, along with spasticity. His Glasgow Coma Scale (GCS) score was 7/15, and he was subsequently intubated. His past medical history included multiple strokes at the age of 14 and another event at age 20, resulting in residual weakness with subsequent improvement. A CT scan of the brain showed IVH with minimal SAH, along with previously known right and left parietal infarctions (Figure 1).

**Figure 2:**
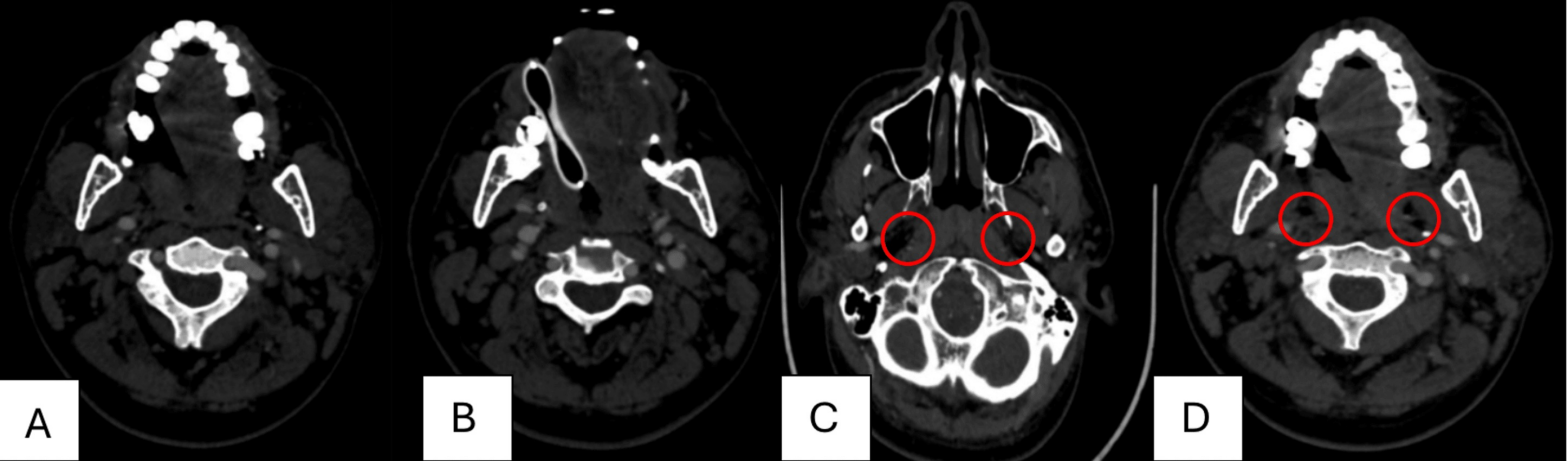
CTA revealed the absence of contrast filling in both ICAs at the level of bifurcation (red circles in C and D) CTA: computed tomographic angiography; ICA: internal carotid artery

The patient was loaded with intravenous phenytoin (1g IV) for seizure prophylaxis. Computed tomographic angiography (CTA) revealed the absence of contrast filling in both ICAs at the level of bifurcation, with prominent posterior circulation, as illustrated in Figure 2.

**Figure 3:**
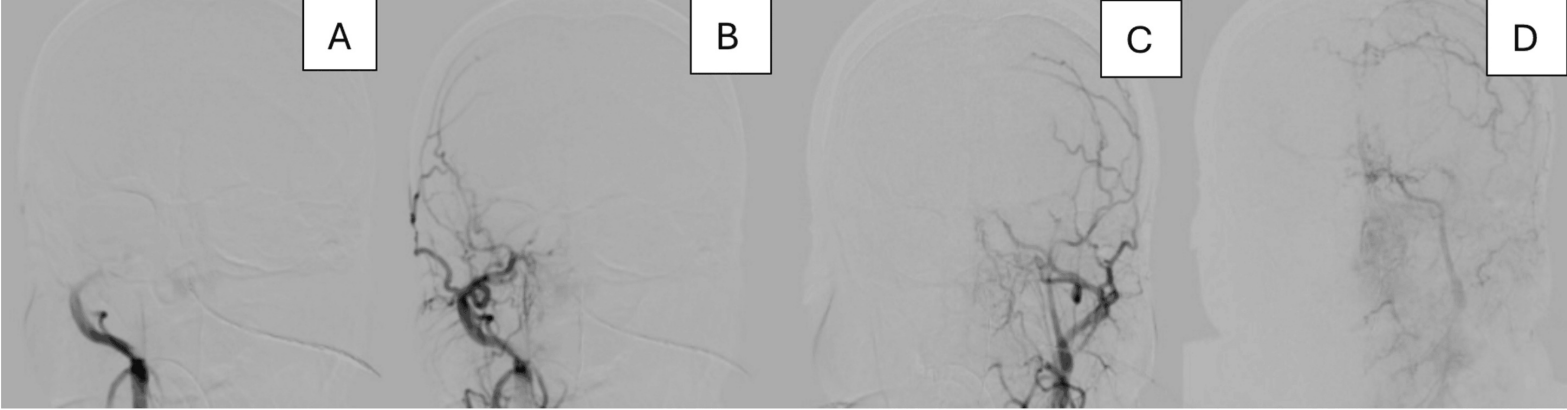
DSA of both common carotid arteries. A and B reveal complete absence of the right ICA, while C and D reveal near-complete occlusion of the left ICA, terminating at the carotid terminus DSA: diagnostic cerebral angiography; ICA: internal carotid artery

A diagnosis of MMS was suspected. The patient was then taken to the angiography suite for diagnostic cerebral angiography (DSA) to rule out vascular causes of spontaneous intraventricular bleeding. Injection of both common carotid arteries revealed a complete absence of the right ICA and almost complete occlusion of the left ICA, terminating at the carotid terminus (Figure 3).

**Figure 4:**
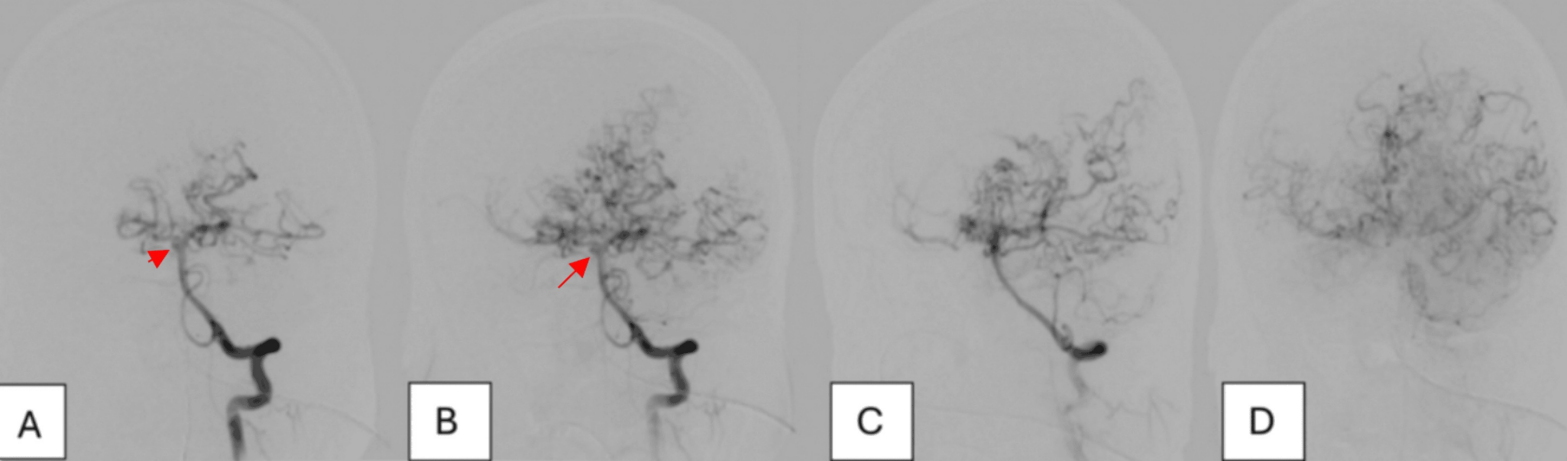
(A-D) Vertebral artery angiogram revealing classic moyamoya collateral vessels (“puff of smoke” appearance) and a right basilar/SCA flow-related aneurysm (red arrow) SCA: superior cerebellar artery

Vertebral artery injection revealed the classic “puff of smoke” appearance, with collateral vessels to the ACA and MCA territories, along with a right basilar/superior cerebellar artery (SCA) flow-related aneurysm (red arrow) (Figure 4).

The IVH with minimal SAH is thought to be related to collateral vessel rupture, as the location of the collaterals corresponds to the pattern of bleeding rather than the small unruptured aneurysm.

The patient was admitted to the intensive care unit (ICU) and managed conservatively according to the NASAH protocol. His hospital course was complicated by multiple strokes, and the patient eventually passed away.

## Discussion

The classic clinical presentation of MMD ranges from ischemic attacks to hemorrhagic events, most frequently ventricular and/or parenchymal hemorrhage. This presentation is due either to rupture of fragile moyamoya vessels or to ruptured aneurysms, both of which can be identified on digital subtraction angiography (DSA) [17].

SAH associated with MMD is mainly caused by rupture of aneurysms, predominantly in the posterior circulation, which are at high risk of rupture due to increased hemodynamic stress. The development of aneurysms in the posterior circulation is related to the significant role of the vertebrobasilar system in providing collateral circulation in MMD, which increases hemodynamic stress and contributes to aneurysm rupture [17].

Nevertheless, the presentation of NASAH in adult patients with MMS is extremely rare. To our knowledge, only 10 cases have been previously reported, which are summarized in Table 1 [8-16].

**Table 1:** Reported cases of NASAH in patients with MMD MMD: moyamoya disease; NASAH: non-aneurysmal subarachnoid hemorrhage; SAH: subarachnoid hemorrhage

Author	Gender	Age, y	Clinical symptoms	Site of bleeding	Etiology of hemorrhage	Associated condition
Dietrichs E, et al. [9]	Female	21	Headache, confusion, seizure, and right hemiparesis	Left frontal and parietal cortex and left Sylvian fissure	Rupture of small, dilated moyamoya vessels	Post-partum
Marushima A, et al. [11]	Female	38	Headache and nausea	Left frontal cortex	Rupture of fragile transdural anastomotic vessels	None
Sönmez G, et al. [14]	Male	31	Loss of consciousness, right hemiparesis, and disorientation	Left frontal and parietal cortex and left Sylvian fissure	Rupture of fragile collateral vessels	Heroin addiction
Osanai T, et al. [13]	Female	34	Headache and seizure	Left frontal cortex	Rupture of dilated collateral arteries on the brain surface	None
Matsumoto Y, et al. [12]	Female	32	Numbness of the left hand and speech disturbance	Right interhemispheric parietal cortex	Undetermine	Postpartum and renal artery stenosis
Fujimura M, et al. [10]	Male	59	Asymptomatic, with multiple TIA episodes	Interhemispheric cistern	Undetermined	Hypertension
Alcala-Cerra GA, et al. [8]	Male	53	Severe headache with dysarthria, altered state of consciousness, and syncope	Left frontal and parietal cortex	Rupture of fragile moyamoya vessels	None
Wu H, et al. [17]	Male	48	Headache and altered mental state	Basal cistern, left Sylvian and longitudinal fissure, left lateral ventricle, and left frontoparietal cortex	Rupture of fragile moyamoya vessels and transdural anastomotic vessels	None
Toscano M, et al. [16]	Female	57	Headache followed by a partial seizure	Right frontal cortex and parieto-occipital cortex bilaterally	Undetermined	None
Mostafa MA, et al. [15]	Female	34	Headache and altered level of consciousness	Bilateral diffuse SAH	Rupture of fragile moyamoya vessels	None

It is believed to be due to the rupture of fragile transdural collateral vessels, potentially with direct extension via perivascular spaces, as seen in our case.

Previously reported cases have shown a higher occurrence in the left frontal cortex, suggesting rupture of transdural anastomotic vessels that traverse the subdural and subarachnoid spaces, lack structural support, and are therefore vulnerable to damage from even minor head trauma [8].

Dietrichs et al. and Matsumoto et al. each documented two instances of NASAH in patients with MMD during the postpartum period. This indicates that hemorrhagic cerebrovascular events are more common during this period, as well as during pregnancy, and may even serve as the initial manifestation of the condition [9,12].

Diagnosis is challenging, often leading to patients reaching an advanced stage without a timely diagnosis. The key to managing these patients is early identification of the disease so that appropriate management can prevent fatal sequelae before a terminal stage is reached. The typical angiographic features were first described in 1957 as hypoplasia of the bilateral ICAs and were later termed the “puff of smoke” appearance by Suzuki and Takaku in 1969 [1]. They classified disease severity based on the degree of ICA narrowing or occlusion and the presence of collateral vessels into six grades to help guide clinical management [1].

A management plan must be implemented once the diagnosis has been established and the patient has been stabilized, particularly in cases presenting with intracranial hemorrhage. Medical management with dual antiplatelet therapy is initiated in patients presenting with ischemic stroke, along with strict blood pressure control to ensure adequate cerebral perfusion.

In cases of NASAH, the standard of care is conservative medical management following SAH protocols. Surgical revascularization using various techniques may also be considered, either direct or indirect, depending on anatomical and patient-specific factors. The most commonly used direct revascularization technique is superficial temporal artery-to-middle cerebral artery bypass, while encephaloduroarteriosynangiosis is a well-established indirect revascularization procedure. In cases of SAH due to ruptured aneurysms associated with high-flow collaterals, usually in the posterior circulation, emergency endovascular treatment is required to prevent life-threatening rebleeding [18].

## Conclusions

Our presented case aligns with previously reported cases, providing further evidence to support the idea that NASAH in MMD is caused by both moyamoya vessel rupture and rupture of transdural anastomotic vessels. Early recognition is crucial; SCD patients with a history of ischemic stroke should be screened for moyamoya to allow timely management and reduce the risk of further neurological decline.
